# Transcriptome- and proteome-wide association of a recombinant inbred line population revealed twelve core QTLs for four fruit traits in pepper (***Capsicum annuum*** L.)

**DOI:** 10.1093/hr/uhac015

**Published:** 2022-02-11

**Authors:** Zhoubin Liu, Bozhi Yang, Renyan Huang, Huan Suo, Zhuqing Zhang, Wenchao Chen, Xiongze Dai, Xuexiao Zou, Lijun Ou

**Affiliations:** 1 Engineering Research Center of Education Ministry for Germplasm Innovation and Breeding New Varieties of Horticultural Crops, College of Horticulture, Hunan Agricultural University, Changsha 410125, China; 2 Plant Protection Institute of Hunan Academy of Agricultural Science, Changsha 410125, China; 3 Vegetable Institution of Hunan Academy of Agricultural Science, Changsha, 410125, China

Dear Editor,

Pepper (*C. annuum* L.) is an important vegetable crop worldwide [1], with remarkable diversity in morphology, nutrition, color, flavor, and yield. A great effort has been made to identify quantitative trait loci (QTLs) and genes that affect these traits [2, 3]. However, few effective combinatorial events in small population sizes have limited the mapping of high-confidence QTLs for marker-assisted breeding (MAB) and map-based gene cloning. The combined multi-omics approach has provided powerful tools for the rapid mining of candidate genes for MAB and for improving our understanding of candidate genes and their molecular regulation [4, 5].

To construct a pepper recombinant inbred line (RIL) population, we generated an F_1_ generation by crossing a late-maturing solitary pod pepper (16 L816) with a late-maturing sweet pepper (16 L15) and then selfed the F_1_ generation for an additional six rounds (Fig. S1). We cultivated the resultant 148 RIL plants and collected data on four important fruit-related traits: fruit color (FC), fruit width (FWD), fruit length (FL), and fruit weight (FWT). These four traits varied considerably among independent lines. Correlation analysis indicated that FWD had a significant positive correlation with FWT and a significant negative correlation with FL (Table S1).

By performing RNA sequencing, RNA-seq analysis, and SNP marker development on fruits from all 148 RIL progenies, we obtained high-quality bin markers consisting of 78,605 SNPs and divided them into 12 linkage groups (LGs) corresponding to 12 chromosomes (Fig. 1a). These SNPs were used to construct a high-density genetic linkage map for pepper. The total genetic distance of the map was 1737 cM, with an average distance of 0.73 cM between adjacent SNPs (Table S2). By comparing the genetic map with physical locations in the reference genome, we found that most segments had high collinearity, although a small number of abnormalities may have been caused by chromosome assembly errors (Fig. 1b). We used the R package qtl [6] to perform interval mapping with the expectation–maximization algorithm (EM) and to calculate the logarithm of odds (LOD). Genome-wide QTL analysis was performed using the high-density linkage map and the phenotypic data on four traits obtained from the RIL population. LOD scores above 3.3 were used to define effective QTLs, and we identified twelve significant QTLs for the four pepper traits (Fig. 1c, Table S3). One QTL for FC (*qFC6.1*) was located on LG 6 and contained 51 candidate genes, including *capsanthin-capsorubin synthase* (*CCS*) (*Capana06g000615*) [7]. There were two significant QTLs for FL (*qFL3.1* on LG 3 and *qFL7.1* on LG 7), four QTLs for FWD on LGs 2, 3, 6, and 11, and five QTLs for FWT (*qFWT2.1* on LG 2, *qFWT4.1* on LG 4, *qFWT6.1* on LG 6, *qFWT7.1* on LG 7, and *qFWT7.2* on LG 7).

**Figure 1:**
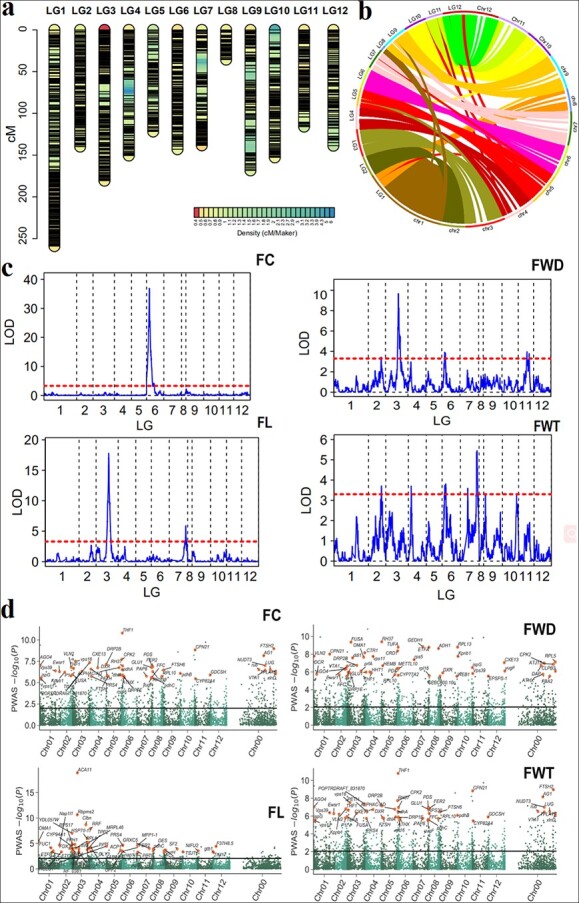
Transcriptome- and proteome-wide association of the recombinant inbred line population. (a) High-density genetic map of pepper. (b) Collinearity analysis of the genetic map with the physical positions of the reference genome. (c) Genome-wide scanning of QTLs for pepper fruit traits. The red lines indicate the significance threshold, with a LOD score of 3.3. Inner ticks on the x-axis depict the locations of observed markers. (c) Manhattan plot of TWAS for fruit traits. The black line represents the significance threshold calculated by 1000 permutations. FC, fruit color; FWD, fruit width; FL, fruit length; FWT, fruit weight.

To identify the genes that regulate these traits, we carried out transcriptome-wide association studies (TWAS) on all RILs to detect correlations between gene expression and phenotype using the limix module [8]. Among the 26 632 genes that passed quality control, 421,021, 1494, and 1594 significantly expressed genes (FDR < 0.05) were detected for FC, FWD, FL, and FWT, respectively (Fig.S2). By comparing the physical locations of all genes on the LG with the eQTL interval, we defined genes whose start interval intersected with the eQTL interval as *cis*-eQTLs and others as *trans*-eQTLs [9]. The expression levels of 2700 of the 6158 genes were regulated by *cis*-eQTLs, and those of 4082 genes were regulated by *trans*-eQTLs. A total of 574 genes had multiple *trans*-eQTLs, resulting in the detection of 4710 *trans* eQTLs, accounting for 63.6% of all eQTLs. Nine trans-eQTL hotspots were detected on the 12 LGs using the qtlhot package (https://CRAN.R-project.org/package=qtlhot) (LOD 3.43 and significance level of 0.01–0.1). They were located on LGs 1, 2, 3, 4, 6, 7, 9, 11, and 12, with LG6–1 containing a total of 645 genes.

We performed label-free proteome sequencing of all fruits in the RIL population and detected 7923 peptides, 7047 of which could be quantified. Based on proteome-wide association analysis (PWAS) of these peptides, 864 proteins were significantly associated with FC, and 855, 304, and 811 proteins were significantly associated with FWD, FL, and FWT, respectively (Fig. 1d). A similar pQTL analysis of the proteomic data revealed that 26.7% of the proteins were significantly associated with *cis* pQTL sites and 73.2% of those were significantly associated with *trans*-acting sites. Seventeen protein hotspots coincided with eQTLs. The hotspot locus LG6–1 contained 455 proteins, suggesting that the LG6–1 region has complex regulatory relationships at both transcriptional and translational levels during pepper fruit development.

To investigate the regulatory mechanisms of pepper fruit traits using multi-omics data, we performed a joint multiple histology analysis. We first identified the overlap of genes significantly associated with each trait in TWAS and PWAS and found that 37 genes were significantly associated with all four traits in the PWAS analysis (Table S4, Fig.S3), suggesting that some common regulatory pathways control these traits. We also compared the overlap of significant genes between TWAS and PWAS for each trait, and 2, 21, 15, and 42 overlapping genes were identified for FC, FWD, FL, and FWT, respectively (Table S5, Fig.S3). However, few genes overlapped between TWAS and PWAS for more than one trait.

In addition to PWAS and TWAS, we also used metabolic pathway analysis to successfully localize the pepper fruit color gene *CCS.* Combined with the results of PWAS and TWAS, this approach enabled us to identify the upstream carotenoid biosynthesis genes *1-Deoxy-D-xylulose 5-phosphate reductoisomerase* (*DXR*) and *phytoene desaturase* (*PDS*) in the RIL population; these genes may also play an important role in the regulation of pepper fruit color.

In the present study, 12 significant QTL loci for pepper FC, FWD, FL, and FWT, and 80 significant trait-associated genes were identified through QTL mapping in an RIL population and association analysis of traits using multiple transcriptional and translational data. We used a relatively small population of RIL lines to effectively associate QTLs and important genes by TWAS and PWAS analysis. Integrated omics analyses can help us to understand the regulatory mechanism of pepper fruit development and to accelerate the mining and cloning of candidate genes. The QTLs detected in this study will be useful for selecting appropriate RILs for further marker-assisted selection when breeding hybrid peppers of diverse market types.

## Supplementary Material

Web_Material_uhac015

## Data Availability

The raw data required to reproduce these findings cannot be shared at this time as they also form part of an ongoing study.
