# Supplementary material for: Transcriptome- and proteome-wide association of a recombinant inbred line population revealed twelve core QTLs for four fruit traits in pepper (Capsicum annuum L.)
Source: Hortic Res. 2022 Feb 11;9:uhac015. doi: 10.1093/hr/uhac015 (PMC9016867; doi:10.1093/hr/uhac015)
Supplement: Web_Material_uhac015 [file web_material_uhac015.docx]

**Supplementary Document S1**

**This document includes the following contents:**

**Page 2-6: Supplementary Materials and Methods**

**Page 7-8: References for Supplementary Materials and Methods**

**Page 9-17: Supplementary Figures and Legends.**

**Page 9: Fig. S1:** **Plant phenotype and study design of RIL population.**

**Page 10-11: Fig. S2:** **Manhattan plot of TWAS for fruit traits.**

**Page 12: Fig. S3: Intersection analysis of candidate genes for different types of analysis.**

**Page 13: Table. S1: Correlation coefficients between fruit-related traits of pepper fruits.**

**Page 14: Table. S2: Summary of the linkage map of pepper fruits.**

**Page 15: Table. S3: Identified QTL for pepper fruit traits.**

**Page 16: Table. S4: Overlapped significant genes of PWAS for Capsicum annuum L. in fruit color (FC); fruit width (FWD); fruit length (FL) and fruit weight (FWT).**

**Page 17: Table. S5: Overlapped genes for TWAS and PWAS.**

**METHODS**

**Plant materials, collection of phenotype data**

F_1_ was obtained by crossing a late-maturing solitary pod pepper (16L816) as a female parent and a late-maturing sweet pepper (16L15) as a male parent, and F_2_ was obtained by self-crossing F_1_. Then we randomly selected 148 single plants in F_2_ segregation populations to seed, and self-crossing of every single plant generated a line. The line was retained a single plant to self consecutively to get the 7^th^ generation. Each line of the 7th generation was sown with 10 plants, and there was no difference in leaf morphology. Seeds were sown in 10 cm × 10 cm plastic pots with the nutrient substrate and grown in a greenhouse (16 h of light at 30 ± 2 ^o^C and 8 h of darkness at 20 ± 2 ^o^C). At the ripening stage of pepper fruit, we took 2 cm long samples of pepper flesh with removed seeds, each sample was collected from one plant and three biological replicates were collected for each RIL strain. About the FWD, FL and FWT traits, we selected 10 pepper fruit ripening to measure the total width and average length. Afterward, the total weight of the 10 pepper fruit ripening was measured and the final average value of each pepper fruit was obtained as the weight of the pepper. For FC, we observed the fruit color on each material with the naked eye and recorded it.

**RNA sequencing**

For RNA sequencing, three biological replicates in each RIL line were performed. Total RNA samples were qualitatively controlled using an Agilent 2100 Bioanalyzer and used for subsequent experiments after qualification. The mRNA was enriched with Oligo dT beads, fragmented, and then reverse transcribed into cDNA using N6 random primers. Subsequently, using the synthesis of cDNA duplexes to form double-stranded DNA. After PCR, the product was heat-denatured to single-stranded, and then a bridge primer was used to loop the single-stranded DNA to obtain a single-stranded circular DNA library for sequencing.

**Genetic linkage map construction**

We obtained an average of 43 M raw reads for each line by above RNA sequencing. The raw reads were filtered using fastp^1^ to get the clean reads, and then the clean reads were aligned to the pepper genome (Zunla-1 version)^2^ using bwa^3^, and with an average alignment rate of 89.6%. GATK^4^ was used to identify population SNPs and filtered using bcftools^5^ with the suggested parameters from GATK. Finally, we obtained a total of 78,605 SNPs in the 148 RILs and parents (X16L15 and X16L816). Then, we selected all homozygous but different sites (aa×bb) between two parents based on the genotyping as polymorphic markers. And next, we extracted the genotypes of 148 progeny at the parental polymorphic marker sites. SNPbinner^6^ software was used to construct the bin map of each RILs. The Hidden Markov Model (HMM) was used to calculate the recombination breakpoints on each chromosome and infer the recombination breakpoint. After that, all high-quality SNP markers allocated into 12 linkage groups (LGs) were constructed a final genetic linkage map. And the genetic distance between adjacent markers was estimated using the Kosambi (1943) mapping function^7^.

**QTL analysis**

The R/qtl package^8^ was used to perform QTL mapping analysis for the RIL population, the three continuous phenotype (FWD,FL,FWT) data were transformed using a logit function as final phenotypes for QTL mapping. First, we use scanone method to map all chromosomes (interval mapping, IM). Using the composite interval mapping (CIM) method, additional linked QTLs were detected by the addqtl function performed forward and backward selection and the elimination algorithm using the stepwiseqtl function. Subsequently, the fitqtl function is used to select the variance analysis regression model that can best explain the difference in phenotypic distribution. And 1000 permutation tests and significance index alpha of 0.05 were used to screen significant QTL. Finally, we calculated the confidence interval of each QTL as the final QTL interval.

**Protein Extraction**

The sample was ground by liquid nitrogen into cell powder and then transferred to a 5-mL centrifuge tube. After that, four volumes of lysis buffer (8 M urea, 1% Triton-100, 10 mM dithiothreitol, and 1% Protease Inhibitor Cocktail) were added to the cell powder, followed by sonication three times on ice using a high-intensity ultrasonic processor (Scientz). (Note: For PTM experiments, inhibitors were also added to the lysis buffer, e.g. 3 μM TSA and 50 mM NAM for acetylation.) The remaining debris was removed by centrifugation at 20,000 g at 4°C for 10 min. Finally, the protein was precipitated with cold 20% TCA for 2 h at -20°C. The supernatant was discarded after centrifugation at 12,000 g 4°C for 10 min. The remaining precipitate was washed with cold acetone three times. The protein was redissolved in 8 M urea and the protein concentration was determined with BCA kit according to the manufacturer’s instructions.

**LC-MS/MS Analysis**

The tryptic peptides were dissolved in 0.1% formic acid (solvent A), directly loaded onto a homemade reversed-phase analytical column (15-cm length, 75 μm i.d.). The gradient was comprised of an increase from 6% to 23% solvent B (0.1% formic acid in 98% acetonitrile) over 26 min, 23% to 35% in 8 min and climbing to 80% in 3 min then holding at 80% for the last 3 min, all at a constant flow rate of 400 nL/min on an EASY-nLC 1000 UPLC system.

The peptides were subjected to NSI source followed by tandem mass spectrometry (MS/MS) in Q ExactiveTM Plus (Thermo) coupled online to the UPLC. The electrospray voltage applied was 2.0 kV. The m/z scan range was 350 to 1800 for a full scan, and intact peptides were detected in the Orbitrap at a resolution of 70,000. Peptides were then selected for MS/MS using NCE setting as 28 and the fragments were detected in the Orbitrap at a resolution of 17,500. A data-dependent procedure that alternated between one MS scan followed by 20 MS/MS scans with 15.0s dynamic exclusion. Automatic gain control (AGC) was set at 5E4. Fixed first mass was set as 100 m/z.

**Database Search**

The resulting MS/MS data were processed using Maxquant^9^ (v.2.0.3.0). Tandem mass spectra were searched against *C. annuum* CM334 database concatenated with reverse decoy database. Trypsin/P was specified as a cleavage enzyme allowing up to 4 missing cleavages. The mass tolerance for precursor ions was set as 20 ppm in the first search and 5 ppm in Main search, and the mass tolerance for fragment ions was set as 0.02 Da. Carbamidomethyl on Cys was specified as fixed modification and acetylation modification and oxidation on Met were specified as variable modifications. FDR was adjusted to < 1% and the minimum score for modified peptides were set > 40.

**eQTL and pQTL analysis**

All quantitatively obtained transcripts were first processed, and 35,336 transcripts which expression was greater than 10 CPM in at least 1/4 of all offspring samples were screened for e-trait, and then the quantitative values were standardized using the DESeq2 package^10^ in R. And all proteins were used for pQTL scan. For eQTL and pQTL analysis, using the scanone function in the qtl package of the R to perform interval mapping and the expectation-maximization (EM) algorithm. And the randomly selected e-trait is tested with 10,000 permutations to obtain the LOD threshold of 3.43 and 3 at the 0.05 significance level for mRNA and protein, then to screen genome region that LOD values were greater than a LOD threshold as candidate eQTLs and pQTL. for each eQTL and pQTL, the 95% Bayesian confidence interval was calculated using the bayesint function in the qtl package and the interval replaced with the reference genome (Zunla-1 version2) was converted to the physical location of each linkage group (LG) in the genome. The physical interval of each eQTL or pQTL on the LG was obtained by combining the start and end positions of each variant frame marker on the chromosome. In addition, since the average physical distance between all markers was 0.1 Mb, we extended each eQTL interval by 0.05 Mb at both ends. By comparing the physical location of all genes on the LG with the eQTL or pQTL interval, we defined the genes which located in the eQTL or pQTL internally interval as cis-eQTL/cis-pQTL, and the others as trans-eQTL/trans-pQTL. qtlhot package in R for trans-eQTL hotspot probing, using the hotperm function for 100 replacement tests, lod.thrs for eQTL analysis selected 0.05 significance level LOD value of 3.43, alpha significance level set to 0.01-0.1, drop. lod N was 47 at the 0.05 significance level at LOD=3.43.

**References for Supplementary Materials and Methods**

1. Chen, S., Zhou, Y., Chen, Y. & Gu, J. Fastp: an ultra-fast all-in-one Fastq preprocessor. *Bioinformatics* **34**, i884-i890 (2018).
2. Qin, C. et al. Whole-genome sequencing of cultivated and wild peppers provides insights into Capsicum domestication and specialization. *Proc Natl Acad Sci U S A* **111**:5135-5140 (2014).
3. Li, H. & Durbin, R. Fast and accurate short read alignment with Burrows-Wheeler transform. *Bioinformatics* **25**, 1754-1760 (2009).
4. McKenna, A. et al. The genome analysis toolkit: a mapReduce framework for analyzing next-generation DNA sequencing data. *Genome Res.* **20**, 1297-1303 (2010).
5. Danecek, P. & McCarthy, S.A. BCFtools/csq: haplotype-aware variant consequences. *Bioinformatics* **33**, 2037-2039 (2017).
6. Gonda I, Ashrafi H, Lyon D A et al. Sequencing-Based Bin Map Construction of a Tomato Mapping Population, Facilitating High-Resolution Quantitative Trait Loci Detection. *Plant Genome*, 2019, **12**
7. Kosambi D. The estimation of map distances from recombination values. *Ann Eugenics* **12**, 172–5 (1943).
8. R/qtl: QTL mapping in experimental crosses. *Bioinformatics* **19**, 889–890 (2003).
9. Prianichnikov N, Koch H, Koch S et al. MaxQuant Software for Ion Mobility Enhanced Shotgun Proteomics. *Mol Cell Proteomics* **19**, 1058-1069 (2020)
10. Wang, L., Feng, Z., Wang, X., Wang, X. & Zhang, X. DEGseq: an R package for identifying differentially expressed genes from RNA-seq data. *Bioinformatics* **26**, 136-138 (2010).

**Supplementary Figures and Legends**


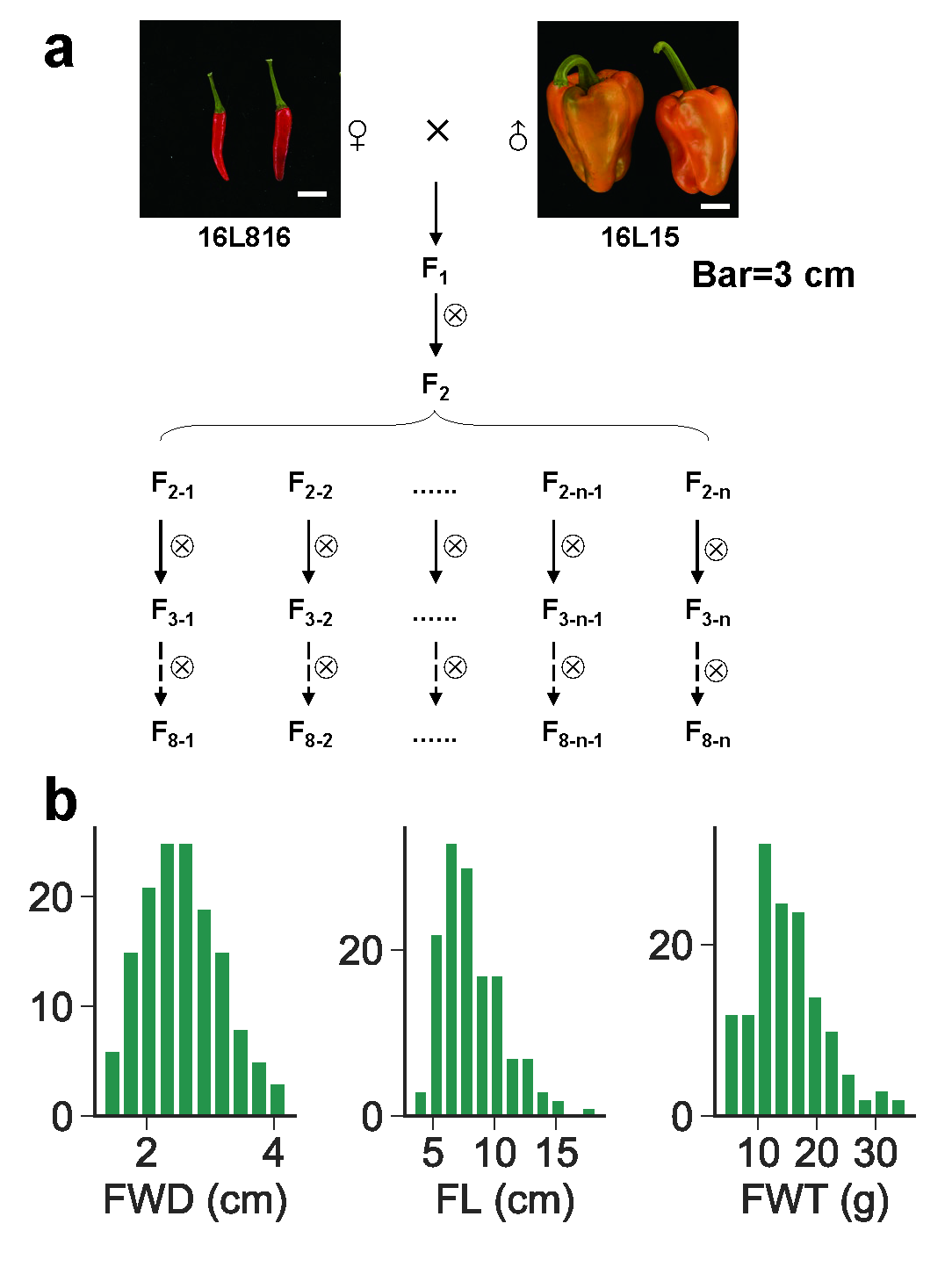


**Fig.S1 Plant phenotype and study design of RIL population**


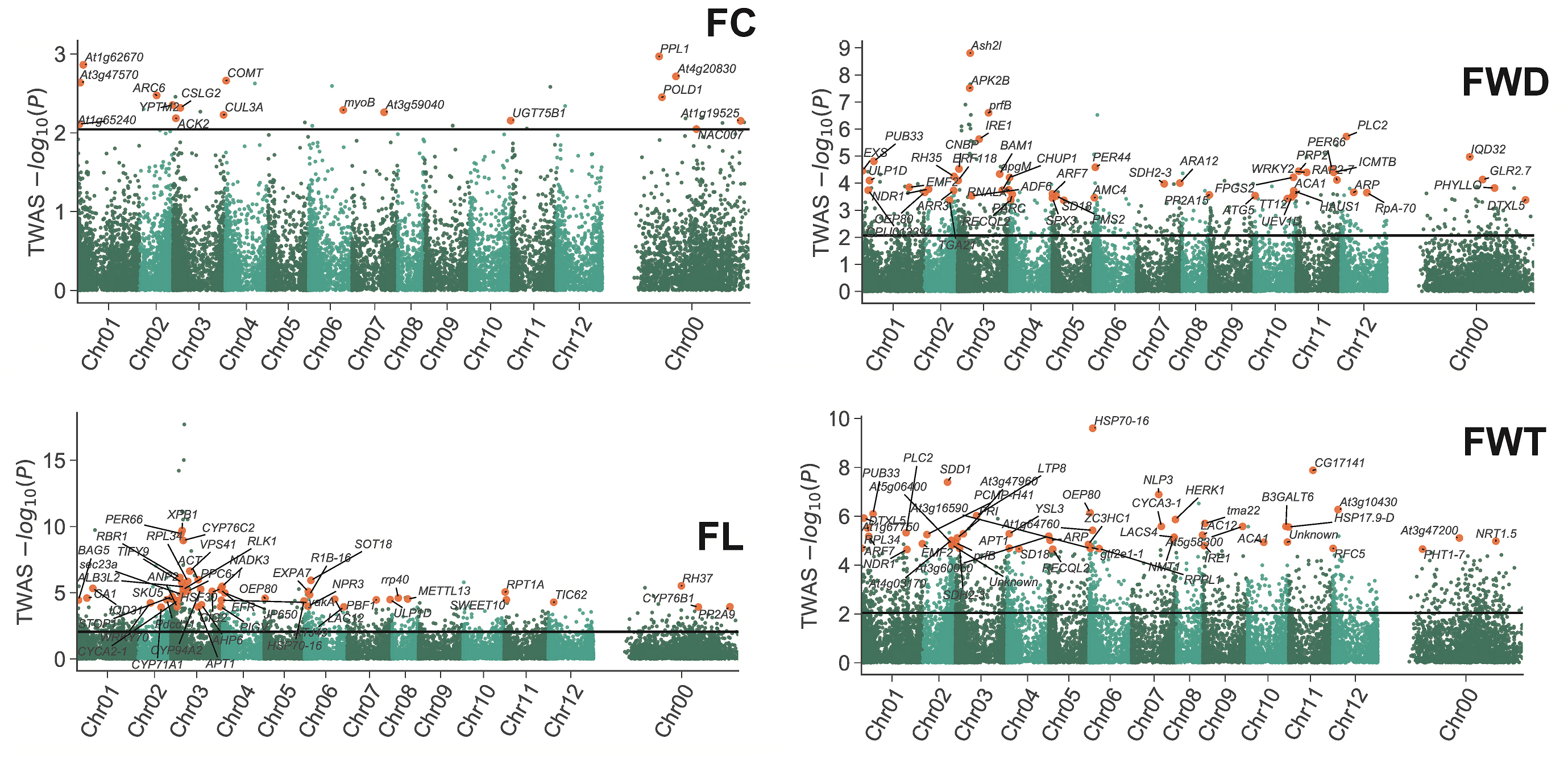


**Fig.S2** **Manhattan plot of TWAS for fruit traits. The black line represents the significance threshold calculated by 1000 times permutation**


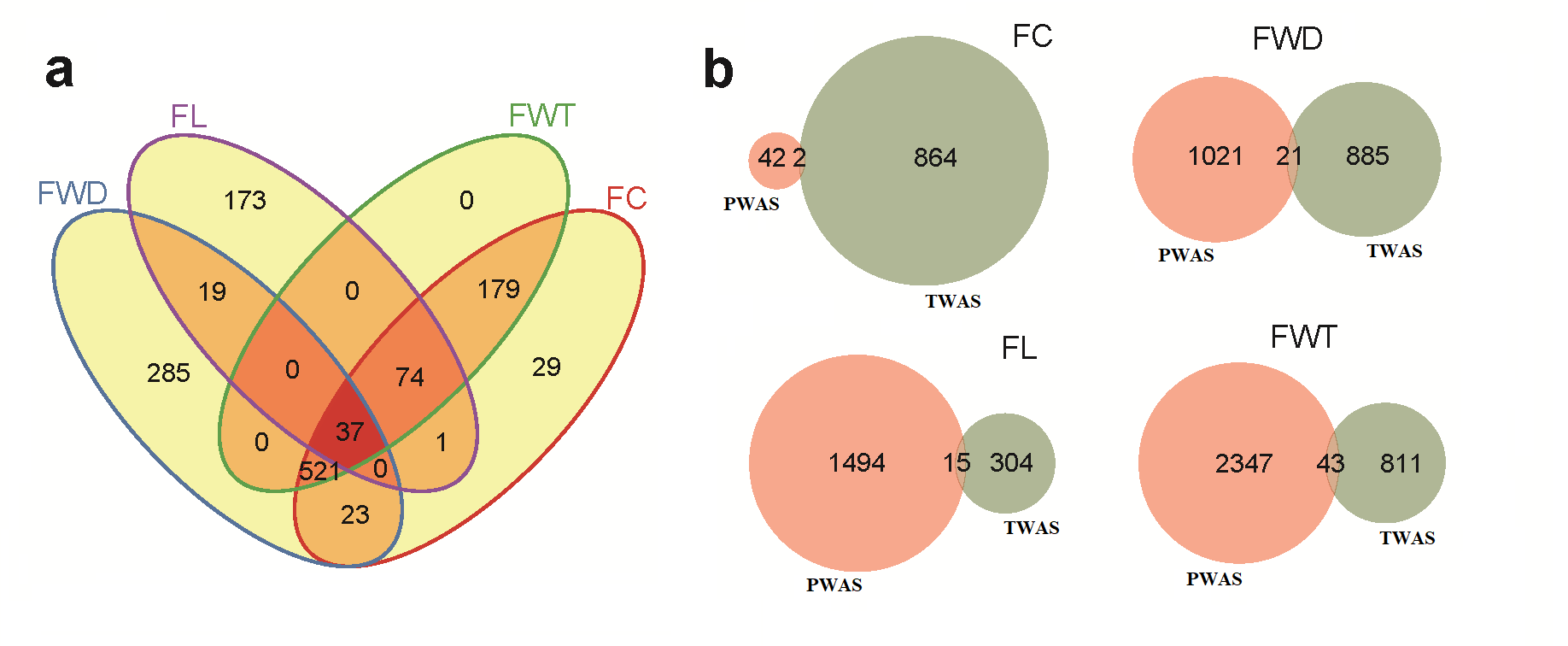


**Fig.S3 Compare analysis of candidate genes for different types of data.**

**Supplementary Tables and Legends**

**Table S1 Correlation coefficients between fruit-related traits of pepper fruits.**

|  | FWD | FL |
| --- | --- | --- |
| FL | -0.12 |  |
| FWT | 0.80** | 0.38** |

**Table S2 Summary of the linkage map of pepper fruits.**

| Chromosome | Linkage Group | Length (cM) | Marker number |
| --- | --- | --- | --- |
| chr1-1,chr8 | LG1 | 259 | 375 |
| chr2 | LG2 | 139 | 224 |
| chr3 | LG3 | 180 | 288 |
| chr4 | LG4 | 150 | 169 |
| chr5 | LG5 | 121 | 134 |
| chr6 | LG6 | 142 | 235 |
| chr7 | LG7 | 138 | 177 |
| chr1-2 | LG8 | 35 | 59 |
| chr9 | LG9 | 168 | 173 |
| chr10 | LG10 | 152 | 180 |
| chr11 | LG11 | 115 | 186 |
| chr12 | LG12 | 138 | 177 |

**Table S3 Identified QTL for pepper fruit traits.**

| **Trait** | **QTL name** | **Linkage group** | **Start (Mb)** | **End (Mb)** | **Min LOD** | **Max LOD** | **Genetic start (cM)** | **Genetic end (cM)** | **Mapping model** |
| --- | --- | --- | --- | --- | --- | --- | --- | --- | --- |
| FC | *qFC6.1* | 6 | 9.2 | 10.0 | 35.80 | 36.94 | 21.71 | 22.43 | scanone |
| FWD | *qFWD2.1* | 2 | 147.9 | 151.0 | 7.39 | 9.17 | 91.75 | 98.02 | cim |
| FWD | *qFWD3.1* | 3 | 64.9 | 73.6 | 13.62 | 15.39 | 94.32 | 95.37 | cim |
| FWD | *qFWD6.1* | 6 | 8.6 | 10.2 | 3.92 | 5.94 | 19.52 | 23.83 | cim |
| FWD | *qFWD11.1* | 11 | 166.9 | 201.7 | 2.07 | 3.98 | 16.48 | 39.59 | scanone |
| FL | *qFL3.1* | 3 | 69.9 | 78.4 | 31.44 | 32.51 | 95.37 | 96.06 | cim |
| FL | *qFL7.1* | 7 | 213.1 | 215.5 | 7.06 | 8.74 | 21.09 | 25.24 | cim |
| FWT | *qFWT2.1* | 2 | 124.6 | 157.2 | 1.56 | 3.71 | 50.99 | 111.80 | scanone |
| FWT | *qFWT4.1* | 4 | 10.6 | 18.2 | 2.35 | 3.70 | 18.25 | 26.20 | scanone |
| FWT | *qFWT6.1* | 6 | 6.2 | 33.4 | 2.12 | 3.81 | 12.77 | 62.05 | scanone |
| FWT | *qFWT7.1* | 7 | 32.4 | 199.7 | 0.92 | 3.60 | 0 | 42.28 | scanone |
| FWT | *qFWT7.2* | 7 | 211.2 | 215.3 | 4.60 | 5.45 | 18.33 | 24.20 | scanone |

**Table S4. Overlapped significant genes of PWAS for Capsicum annuum L. in fruit color (FC); fruit width (FWD); fruit length (FL) and fruit weight (FWT)**

| **Gene** | **FC** | **FL** | **FWT** | **FWD** | **Symbol** |
| --- | --- | --- | --- | --- | --- |
| Capana02g002877 | 3.75E-07 | 7.55E-04 | 3.75E-07 | 1.37E-04 | At1g06030 |
| Capana01g004099 | 5.23E-07 | 3.83E-03 | 5.23E-07 | 1.72E-05 | PAP8 |
| Capana06g000154 | 5.25E-06 | 3.71E-04 | 5.25E-06 | 5.51E-03 | GRXC5 |
| Capana10g000295 | 1.95E-05 | 4.30E-04 | 1.95E-05 | 4.94E-04 | DES |
| Capana00g002444 | 3.98E-05 | 3.56E-03 | 3.98E-05 | 9.01E-04 | AOC3 |
| Capana06g002440 | 3.54E-05 | 3.02E-04 | 3.54E-05 | 7.10E-03 | At5g46580 |
| Capana03g001262 | 6.94E-07 | 5.38E-03 | 6.94E-07 | 1.22E-04 |  |
| Capana07g000484 | 8.60E-05 | 4.94E-03 | 8.60E-05 | 3.05E-03 | PCBP2 |
| Capana05g000740 | 1.73E-06 | 7.73E-03 | 1.73E-06 | 2.20E-04 | pdhB |
| Capana01g002717 | 2.20E-03 | 5.07E-03 | 2.20E-03 | 5.04E-03 | UBP24 |
| Capana04g001609 | 5.34E-06 | 1.97E-03 | 5.34E-06 | 2.40E-03 | pdhC |
| Capana09g001791 | 2.57E-09 | 4.82E-03 | 2.57E-09 | 1.30E-06 | Unknown |
| Capana11g002272 | 1.26E-06 | 4.06E-04 | 1.26E-06 | 5.13E-04 | pdhC |
| Capana03g000562 | 1.64E-08 | 7.85E-04 | 1.64E-08 | 3.66E-04 |  |
| Capana09g000293 | 6.74E-06 | 2.17E-03 | 6.74E-06 | 2.96E-03 | AIM1 |
| Capana06g000748 | 1.35E-06 | 2.67E-03 | 1.35E-06 | 6.65E-05 | RPS30 |
| Capana08g000039 | 1.35E-07 | 7.14E-04 | 1.35E-07 | 1.60E-04 | POPTRDRAFT_831870 |
| Capana07g001961 | 1.72E-07 | 2.86E-04 | 1.72E-07 | 1.64E-03 | PRS4 |
| Capana03g001979 | 1.13E-05 | 1.64E-05 | 1.13E-05 | 8.13E-03 | PPH1 |
| Capana00g002177 | 1.17E-05 | 4.00E-04 | 1.17E-05 | 5.81E-04 |  |
| Capana05g002051 | 1.26E-06 | 2.22E-04 | 1.26E-06 | 2.09E-03 | FER2 |
| Capana09g000016 | 3.30E-07 | 2.21E-03 | 3.30E-07 | 1.96E-04 | pdhA |
| Capana10g002117 | 3.44E-04 | 1.46E-03 | 3.44E-04 | 3.94E-03 | grpE |
| Capana03g002084 | 2.63E-06 | 3.90E-03 | 2.63E-06 | 3.32E-04 |  |
| Capana00g003367 | 3.47E-07 | 6.09E-03 | 3.47E-07 | 4.51E-05 | THF1 |
| Capana02g002822 | 1.34E-03 | 4.11E-04 | 1.34E-03 | 5.84E-03 |  |
| Capana07g002456 | 5.15E-05 | 4.09E-03 | 5.15E-05 | 3.18E-03 |  |
| Capana00g003284 | 5.33E-06 | 4.63E-03 | 5.33E-06 | 2.71E-04 | cp12 |
| Capana10g001920 | 6.28E-09 | 6.51E-04 | 6.28E-09 | 1.73E-05 |  |
| Capana04g000461 | 1.85E-06 | 6.24E-03 | 1.85E-06 | 2.21E-03 | CYP82A4 |
| Capana08g001599 | 3.16E-05 | 1.29E-03 | 3.16E-05 | 3.87E-03 | lpdA |
| Capana01g003621 | 2.70E-05 | 2.14E-03 | 2.70E-05 | 4.06E-03 | Mcat |
| Capana03g003612 | 2.34E-04 | 8.35E-03 | 2.34E-04 | 3.53E-03 | TOC159 |
| Capana04g002867 | 1.21E-05 | 8.00E-03 | 1.21E-05 | 1.35E-03 | Unknown |
| Capana11g000087 | 1.46E-09 | 5.30E-03 | 1.46E-09 | 1.48E-06 | ispG |
| Capana00g000927 | 7.12E-08 | 7.48E-03 | 7.12E-08 | 9.12E-06 |  |
| Capana04g000110 | 2.60E-07 | 3.34E-03 | 2.60E-07 | 8.76E-05 | FTSH |

**Table S5. Overlapped genes for TWAS and PWAS**

| **Gene** | **TWAS *P*** | **PWAS *P*** | **Trait** | **Symbol** |
| --- | --- | --- | --- | --- |
| Capana03g000905 | 6.54E-03 | 6.52E-04 | FC | PPL1 |
| Capana01g000689 | 2.31E-03 | 6.82E-03 | FC | YPTM2 |
| Capana00g005037 | 1.69E-03 | 5.06E-07 | FWD |  |
| Capana01g001376 | 5.71E-03 | 2.64E-03 | FWD | At4g08900 |
| Capana01g002077 | 2.43E-03 | 1.20E-04 | FWD |  |
| Capana01g003044 | 8.10E-03 | 6.60E-03 | FWD | AAEL006684 |
| Capana01g004460 | 1.28E-03 | 5.75E-03 | FWD | SRR |
| Capana02g001321 | 4.39E-03 | 4.51E-03 | FWD | PSAF |
| Capana02g002186 | 6.52E-03 | 3.08E-03 | FWD | HSP70-16 |
| Capana03g000735 | 8.60E-03 | 6.24E-05 | FWD |  |
| Capana03g000806 | 7.98E-03 | 1.85E-05 | FWD |  |
| Capana03g001893 | 1.25E-07 | 3.35E-05 | FWD |  |
| Capana03g002619 | 4.43E-03 | 4.49E-05 | FWD | At5g05200 |
| Capana03g003538 | 4.51E-03 | 3.11E-05 | FWD |  |
| Capana05g000063 | 4.47E-03 | 4.04E-03 | FWD | EMB2776 |
| Capana05g001070 | 3.43E-03 | 4.18E-03 | FWD |  |
| Capana06g000728 | 6.78E-03 | 6.28E-05 | FWD | At1g09340 |
| Capana07g001996 | 2.83E-03 | 4.33E-03 | FWD | ZC3HC1 |
| Capana07g002457 | 8.45E-03 | 3.94E-03 | FWD |  |
| Capana08g000015 | 4.80E-03 | 4.84E-06 | FWD |  |
| Capana09g000186 | 1.67E-03 | 6.41E-03 | FWD | tma22 |
| Capana10g002076 | 3.04E-03 | 1.27E-03 | FWD | At4g31480 |
| Capana12g001296 | 3.19E-03 | 7.95E-03 | FWD | COX6B-1 |
| Capana01g003296 | 6.39E-03 | 4.22E-03 | FL |  |
| Capana02g003059 | 5.89E-04 | 2.83E-05 | FL | Rbpms2 |
| Capana02g003332 | 4.21E-03 | 4.39E-03 | FL |  |
| Capana03g000672 | 2.14E-03 | 7.98E-03 | FL | gtf2e1-1 |
| Capana03g001569 | 2.56E-04 | 6.81E-04 | FL | Rdh12 |
| Capana03g002619 | 2.29E-07 | 6.68E-03 | FL | At5g05200 |
| Capana03g003711 | 8.19E-03 | 4.89E-03 | FL | SGS3 |
| Capana04g000129 | 1.47E-03 | 3.38E-04 | FL | PETE |
| Capana04g001609 | 1.58E-03 | 1.97E-03 | FL | pdhC |
| Capana05g000667 | 1.68E-03 | 7.65E-03 | FL | UBP12 |
| Capana05g002051 | 8.84E-03 | 2.22E-04 | FL | FER2 |
| Capana06g000049 | 6.17E-04 | 3.46E-03 | FL | RPL27 |
| Capana08g000010 | 7.72E-04 | 8.64E-04 | FL |  |
| Capana08g000039 | 3.40E-03 | 7.14E-04 | FL | POPTRDRAFT_831870 |
| Capana09g000183 | 5.92E-05 | 2.12E-04 | FL | nap1l1 |
| Capana00g005037 | 7.16E-03 | 3.99E-06 | FWT |  |
| Capana01g000017 | 3.44E-03 | 6.94E-04 | FWT | RPL11 |
| Capana01g001202 | 9.00E-03 | 5.81E-03 | FWT | WARS |
| Capana01g002077 | 3.36E-05 | 4.97E-04 | FWT |  |
| Contiue |  |  |  |  |
| Capana01g003049 | 8.08E-04 | 1.56E-05 | FWT | GLN2 |
| Capana01g003385 | 1.43E-04 | 4.17E-07 | FWT | DRP1E |
| Capana02g002186 | 9.71E-06 | 5.77E-05 | FWT | HSP70-16 |
| Capana02g002314 | 1.30E-03 | 1.57E-06 | FWT | FTSH6 |
| Capana02g002576 | 4.74E-03 | 8.32E-04 | FWT | HEXBP |
| Capana02g002621 | 5.17E-03 | 8.73E-04 | FWT | FBA2 |
| Capana02g002688 | 1.50E-03 | 4.02E-03 | FWT |  |
| Capana02g003059 | 5.38E-03 | 5.79E-03 | FWT | Rbpms2 |
| Capana03g000947 | 5.84E-03 | 5.58E-04 | FWT | ENO1 |
| Capana03g001004 | 6.44E-03 | 7.06E-06 | FWT | At5g50100 |
| Capana03g001087 | 2.89E-04 | 1.99E-03 | FWT | HIR1 |
| Capana03g001978 | 1.32E-03 | 3.12E-08 | FWT | Kpnb1 |
| Capana03g002430 | 8.52E-03 | 6.72E-03 | FWT | PGMP |
| Capana03g002890 | 8.89E-03 | 4.65E-07 | FWT | RH37 |
| Capana03g003538 | 1.24E-04 | 3.42E-06 | FWT |  |
| Capana03g003655 | 1.88E-04 | 9.71E-04 | FWT | MRPL46 |
| Capana03g003803 | 1.25E-03 | 6.00E-03 | FWT | PR |
| Capana04g001057 | 5.75E-03 | 6.73E-03 | FWT | CRY1 |
| Capana04g001609 | 6.39E-03 | 5.34E-06 | FWT | pdhC |
| Capana05g000063 | 7.68E-03 | 5.90E-03 | FWT | EMB2776 |
| Capana05g001070 | 2.86E-05 | 5.65E-03 | FWT |  |
| Capana05g001818 | 3.06E-03 | 1.13E-03 | FWT | FTSH5 |
| Capana05g002046 | 1.44E-04 | 2.26E-03 | FWT | CHC1 |
| Capana05g002051 | 5.57E-03 | 1.26E-06 | FWT | FER2 |
| Capana06g000077 | 9.92E-03 | 2.31E-04 | FWT |  |
| Capana06g001249 | 5.81E-03 | 3.68E-04 | FWT | APS1 |
| Capana07g000048 | 5.47E-03 | 6.73E-04 | FWT | psaA |
| Capana07g001878 | 2.17E-04 | 2.79E-03 | FWT | CYP72A1 |
| Capana08g000039 | 1.39E-03 | 1.35E-07 | FWT | POPTRDRAFT_831870 |
| Capana08g000045 | 2.94E-03 | 5.10E-07 | FWT | ATHX |
| Capana08g000356 | 5.95E-03 | 6.60E-04 | FWT | RPL3 |
| Capana08g000903 | 5.51E-03 | 8.05E-03 | FWT |  |
| Capana09g000882 | 3.09E-03 | 2.57E-03 | FWT |  |
| Capana10g002082 | 1.67E-03 | 5.33E-04 | FWT | SYP24 |
| Capana11g000118 | 5.27E-04 | 1.54E-03 | FWT | PBG1 |
| Capana12g000027 | 3.53E-03 | 4.08E-05 | FWT | idnO |
| Capana12g000159 | 2.86E-04 | 5.13E-03 | FWT | AAE7 |
| Capana12g000407 | 8.16E-03 | 8.35E-04 | FWT | TIC55 |
